# Establishing a distributed national research infrastructure providing bioinformatics support to life science researchers in Australia

**DOI:** 10.1093/bib/bbx071

**Published:** 2017-06-30

**Authors:** Maria Victoria Schneider, Philippa C Griffin, Sonika Tyagi, Madison Flannery, Saravanan Dayalan, Simon Gladman, Nathan Watson-Haigh, Philipp E Bayer, Michael Charleston, Ira Cooke, Rob Cook, Richard J Edwards, David Edwards, Dominique Gorse, Malcolm McConville, David Powell, Marc R Wilkins, Andrew Lonie

**Affiliations:** 1 University of Melbourne Melbourne Institute, Carlton Victoria, Australia; 2 EMBL Australia Bioinformatics Resource, EMBL-ABR Hub, Melbourne, Victoria, Australia; 3 Australian Genome Research Facility, Bioinformatics, 1G royal Pde Parkville, Victoria, Australia; 4 University of Melbourne Bio21 Molecular Science and Biotechnology Institute, Metabolomics Platform, Parkville Victoria, Australia; 5 Adelaide, Australia; 6 University of Western Australia, School of Plant Biology, Crawley, Western Australia, Australia; 7 University of Tasmania Menzies Institute for Medical Research, Hobart Tasmania, Australia; 8 James Cook University, College of Public Health, Medical & Vet Sciences, Townsville, Queensland, Australia; 9 University of New South Wales, Sydney, Australia; 10 University of Western Australia, School of Plant Biology, Crawley, Western Australia, Western Australia; 11 Queensland Facility for Advanced Bioinformatics, Brisbane, Queensland, Australia; 12 University of Melbourne Bio21 Molecular Science and Biotechnology Institute, Parkville Victoria, Australia; 13 Monash University, Melbourne, Australia; 14 University of New South Wales, School of Biotechnology and Biomolecular Sciences, Sydney, Australia; 15 University of Melbourne Department of General Practice and Primary Health Care, Melbourne Bioinformatics, Carlton Victoria, Australia

**Keywords:** EMBL-ABR, distributed network, bioinformatics infrastructure, data-driven analysis, bioinformatics service, bioinformatics training, data-driven science, national, capability, services

## Abstract

EMBL Australia Bioinformatics Resource (EMBL-ABR) is a developing national research infrastructure, providing bioinformatics resources and support to life science and biomedical researchers in Australia. EMBL-ABR comprises 10 geographically distributed national nodes with one coordinating hub, with current funding provided through Bioplatforms Australia and the University of Melbourne for its initial 2-year development phase. The EMBL-ABR mission is to: (1) increase Australia’s capacity in bioinformatics and data sciences; (2) contribute to the development of training in bioinformatics skills; (3) showcase Australian data sets at an international level and (4) enable engagement in international programs. The activities of EMBL-ABR are focussed in six key areas, aligning with comparable international initiatives such as ELIXIR, CyVerse and NIH Commons. These key areas—Tools, Data, Standards, Platforms, Compute and Training—are described in this article.

## Introduction

The surge of bioinformatics infrastructures at national and international levels has been nothing short of transformational in the past decade. In a wide range of countries and jurisdictions, efforts to establish and support bioinformatics infrastructure for the life sciences have started, and continue to expand. Examples can be found in Europe, such as the Dutch Techcentre for Life Sciences (DTL) in the Netherlands, the German Network for Bioinformatics Infrastructure (deNBI) and ELIXIR at the European level; in the United States, including the National Institutes for Health’s ‘Big Data to Knowledge’ (BD2K) and the National Science Foundation’s CyVerse; and in Canada with Bioinformatics/Computational Biology Framework (B/CB).

The establishment of ELIXIR has acted as a catalyst for many European countries that either had some existing level of national bioinformatics infrastructure or had to develop this *de novo*. ELIXIR is a distributed infrastructure for life science information which aims to coordinate, integrate and sustain bioinformatics resources across its member states—including those at the European Molecular Biology Laboratory’s European Bioinformatics Institute (EMBL-EBI)—to enable users in academia and industry to access vital data, tools, standards, compute and training services for their research [1]. A different approach is taken in the United States, where life science, agriculture and biomedical researchers can access multiple major bioinformatics projects and resources that act at the national level to provide bioinformatics infrastructure: (a) the National Center for Biotechnology Information (NCBI) advances science and health by providing access to biomedical, agricultural and genomic information [2]; (b) CyVerse is a dynamic virtual organization that provides scientists across all life sciences disciplines with computational infrastructure to handle huge data sets and complex analyses, thus enabling data-driven discovery [3]; (c) the NIH Commons is a virtual platform for biomedical data, software, metadata and workflow discovery, management, sharing and reuse [4]; (d) BD2K is a trans-NIH initiative established to enable biomedical research as a digital research enterprise, to facilitate discovery and support new knowledge and to maximize community engagement [5]; (e) the AgBioData working group aims to coordinate bioinformatics capability and expertise in the United States Department of Agriculture.

Australia, although large geographically, has a relatively small number of bioscientists: 30–35 000 full-time equivalent based on our estimate of 30–35% of the total ∼100 000 Australian researcher workforces [6], compared with ∼500 000 bioscientists in the European Union, which is about half the area of Australia. The Australian biomedical/bioinformatics community is concentrated in several large, but geographically separated research precincts, usually in the major state/national capital cities, and face-to-face meetings between collaborators across states realistically require air travel—typically 1 h (e.g. Melbourne, Sydney or Adelaide) to 4.5 h (Perth–Brisbane or Sydney). Although relatively well temporally aligned with Asian countries [e.g. China (0–2 h) and India (2.5–5.5 h)], Australia has substantial time differences with Europe (9–11 h) and North America (14–16 h). This often means that considerable logistic effort is required for international collaborations, with meetings that are either early in the morning or late at night.

These factors create a set of challenges when it comes to the maintenance of important collaborations at a national or international level, and Australia’s geographic characteristics have of course influenced the development of national infrastructure in support of bioinformatics and biosciences research.

Like many countries, Australia has a long but somewhat inconsistent history of providing national bioinformatics infrastructure, often in the context of numerous institutional and state-based efforts. An early example is the Australian National Genome Information Service [6], started in 1991 but which ceased operations in 2009, which itself evolved out of the Sydney University Sequence Analysis Interface. Shortly after, a Bio-Mirror [7] was established at Australian National University, providing high-speed access to common hosted data sets, which of course was (and remains) an important factor in Australian digital research infrastructure access because of intercontinental network bandwidth constraints. Significant investment in national bioinformatics was made through the Australian Research Council Centre of Excellence in Bioinformatics [8] from 2003 to 2010, which in turn provided a natural context for the establishment of the EBI Mirror project at the University of Queensland in 2010. Concurrently, in 2006, a major infrastructure investment through the National Collaborative Research Infrastructure Scheme (NCRIS) [9] was made in omics data platform infrastructure through Bioplatforms Australia [10], and the Australian Bioinformatics Facility [11], hosted by the Centre for Comparative Genomics at Murdoch University, was established in support of that data infrastructure. As the discipline of bioinformatics grew, several initiatives to form an Australian Bioinformatics Network (ABN) were made in 2006 and 2012 [12] to foster the developing bioinformatics community and its connection to the broader research community; in 2014, ABN evolved into the Australian Bioinformatics And Computational Biology Society (ABACBS) [13] in response to the maturing of bioinformatics in Australia as a discipline. As ABN evolved in support of bioinformatics, so did the EBI Mirror project in support of bioinformatics infrastructure, in 2012, becoming the Bioinformatics Resource Australia of EMBL, Australia, also known as BRAEMBL [14]. The project evolved from this original concept to also encompass services across the areas of tools and training across Australia in 2015. In February 2016 the project was relaunched as a national network of bioinformatics infrastructure services: EMBL Australia Bioinformatics Resource (EMBL-ABR).

Access to hardware, bioinformatics services and data remain a priority in biosciences, and the challenges involved across the biological domains are shared, so implementing solutions at national level means reducing redundancy of effort and ensuring longer-term sustainability for the maintenance and further developments [15–18]. EMBL-ABR is an evolving response towards a federated network of bioinformatics infrastructure in Australia to address these challenges and provide a suitable ‘digital ecosystem’ that leverages on existing NCRIS capabilities such as Research Data Services, the National eResearch Collaboration Tools and Resources (Nectar), Australian National Data Service (ANDS) and BPA and is also linked and immersed in the cutting-edge developments that are taking place overseas.

## EMBL-ABR structure

**Figure 1:**
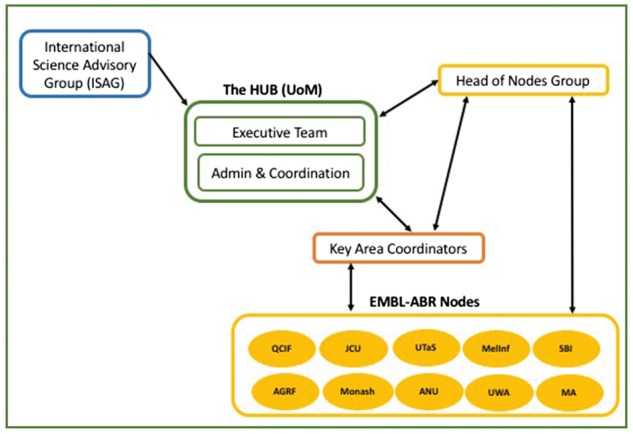
Structure of EMBL-ABR.

EMBL-ABR has a coordinating hub hosted by Melbourne Bioinformatics at the University of Melbourne and 10 nodes across Australia, which represent existing institutions and facilities that are already working in one or more of the six key areas. The overall administration, communication and outreach capacity are provided by the hub. Figure 1 shows the current structure of EMBL-ABR. The scientific remit and actual activities are spread across the nodes, with the aim to form a roadmap to develop bioinformatics resources as well as adopt existing solutions that can be federated in Australia once consistent funding is secured. Each node therefore has compiled a form describing their existing capabilities and how these are scalable at state and national level. Initial node descriptions are all published on EMBL-ABR website [19]. Hence, the overall set of EMBL-ABR activities depends on the actual areas of expertise and bioinformatics resources already present in the nodes.

EMBL-ABR Key Areas (summarized in Table 1) map clearly to the ELIXIR Platform topics: Data, Tools, Compute, Training and Interoperability, the last of which is closely related to the EMBL-ABR Key Area of Standards. These key areas encompass all aspects of bioinformatics across specific research domains, encouraging expertise sharing and reuse for similar problems identified in distinct research domains. EMBL-ABR is also interacting with specific biological domains to address their respective needs, such as wheat annotation and bioinformatics, invertebrate omics and microbial bioinformatics.

**Table 1:** EMBL-ABR Key areas mapped to the ELIXIR platform topics: Data, Tools, Compute, Training and Interoperability

Key area	Aims	EMBL-ABR Nodes working in this Key Area
Training	Provide Hub training on critical topics not covered elsewhere Coordination and dissemination of node end-user training activities (STM, iAnn) Enable improved access to EBI training resources and international experts	All EMBL-ABR Nodes
Data	Provide advice on best practice data management and data life cycle in the Biosciences Showcase Australian data at an international level Catalogue and deliver the bioinformatics data Australia needs	ANU, MA, Melbourne Bioinformatics, QCIF, SBI, UTAS, UWA, AGRF
Standards	Foster adoption of standardized file formats, metadata, vocabularies and identifiers by the Australian Bioscience community Bring Australian needs in standards development to the attention of international efforts Ensure Australian input into development and where appropriate coordination of international standards activities	MA, AGRF, QCIF
Tools	Assess Australian needs for bioinformatics software hosting, maintenance and support Implement a registry of Australian bioinformatics tools, ToolsAU Contribute to the development and dissemination of bioinformatics tools	AGRF, ANU, MA, Melbourne Bioinformatics, SBI, UTAS, UWA
Platforms	Leverage existing expertise in platform development Enable research access to bioinformatics platforms that link multiple tools, facilitate data sharing and access and record analysis pipelines	ANU, JCU, MA, Melbourne Bioinformatics, Monash, QCIF, UTAS, UWA
Compute	Assist in the design, architecture and delivery of compute infrastructure Support network activities to ensure interoperability across Australia and with international efforts	AGRF, Melbourne Bioinformatics, Monash, QCIF

EMBL-ABR Nodes are represented by the Head of the Nodes Group that together with the Hub executive team have a regular monthly meeting based on a shared and collaborative agenda to discuss the strategy as well as actual activities. The Hub executive team is in regular contact with the Head of the Nodes Group and sends updates through its mailing list. The agenda and minutes, plus nodes updates, are also posted online, in the key documents area, so anyone can stay informed and follow-up EMBL-ABR discussions [20].

EMBL-ABR relies on the goodwill and enthusiasm from its nodes and champions and has a group of Key Area Coordinators to collect what the Australian Biosciences needs are for these key areas and aid the development of EMBL-ABR activities and priorities to address such needs. The group has also established monthly meetings to discuss updates as well as solutions and advances in these areas that exist and could be of benefit to federate within Australia.

EMBL-ABR has also established an International Science Advisory Group (ISAG) that brings together 10 members, highly regarded scientists from around the world, to discuss and advise on EMBL-ABR strategy, activities and priorities. EMBL-ABR ISAG is composed of a mix between international bioinformatics infrastructure experts and Australian biological domain experts. The group met face to face in December 2016, and took an active participation in the first EMBL-ABR All-Hands meeting [21]. The review and feedback from the ISAG helps EMBL-ABR to align its strategy with international standards and needs of Australian life science researchers.

EMBL-ABR has direct links with ABACBS through its chair being a member of the EMBL-ABR ISAG, and the EMBL-ABR Training Coordinator being part of ABACBS’s dedicated Education and Training subcommittee.

EMBL-ABR encourages Australian Bioinformaticians to become members of ABACBS, as it offers the best forum to participate and discuss bioinformatics career developments and opportunities and to advance awareness and recognition of this ever-expanding field. ABACBS is also the place for listings for all job opportunities for bioinformaticians across the Australian research community.

## EMBL-ABR mission and remit

EMBL-ABR aspires to:

1. increase Australia’s capacity to collect, integrate, analyse, exploit, share and archive the large heterogeneous data sets, now part of modern life sciences research;
2. contribute to the development and delivery of training in data, tools, platforms and international standards, to enable Australia’s life science researchers to undertake research in the age of big data;
3. showcase Australian research and data sets at an international level; and
4. enable engagement in international programs that create, deploy and develop best practice approaches to data management, software tools and methods, computational platforms and bioinformatics services.

These four objectives contribute overall to create a sustainable bioinformatics infrastructure network that reflects the specific geographical and temporal patterns of those working in Australia.

The life sciences and biomedical bioinformatics communities are critically dependent on the tools needed to store, find, access, annotate, enrich, visualize, integrate and interpret data. Tool development is extremely dynamic, partly because of the fast pace of advancement in data production technologies and the consequent spawning of new file formats. Recent efforts [22–27] aim to describe best practice for academic software. Tools should have exposure, so that redundancy can be avoided, and sustainable plans can be in place for maintenance and/or expansion of functions, as the field and users’ needs evolve. During 2016, EMBL-ABR set up a Tool registry, ToolsAU, powered by the ELIXIR Tools and Data registry [28, 29], which allows users to filter and display the tools created in Australia or with Australian involvement. The current registry (3 October 2016) shows >50 entries for Australian Tools: https://www.embl-abr.org.au/tools/toolsau/. EMBL-ABR is also contributing to the community effort developing open-source principles to promote software quality and sustainability [30]. A Search for Training Material (STM) was implemented to collect Australian training material resources and enhance discoverability of such materials [31].

The quality and utility of data relies on the existence and adoption of standards, shared formats and mechanisms for biological researchers to share and annotate the data, so it can be easily searchable, conveniently linked and consequently used for further biological analysis and discovery [32]. One of the biggest challenges is the inherent heterogeneity of biological data types [32–34]. Effective solutions that reduce the challenges associated with data volume and complexity are being developed worldwide [35, 36]. However, awareness from data producers and consumers of best practice in data management, as well as intuitive tools that allow researchers to easily implement such practices, remains lacking [37–39]. The development of the FAIR principles (Findable, Accessible, Interoperable and Reusable) [40] and recent efforts internationally on applying such principles are critical for ensuring use and reuse [41, 42].

To gather an overview of the spectrum of data Australia contributes in terms of domains in biology, systems and model organisms as well as the type of data, EMBL-ABR launched a BioSharing Collection, so that our quantification of available data would also capture the parameters established by BioSharing and the larger international community in terms of best practice. This is an ongoing activity and as of 3 October 2016 listed 15 databases [43].

EMBL-ABR has also been active in initiating key collaborations for training, standards and federation of services with international partner, including EMBL-EBI, GOBLET, University of Cambridge Bioinformatics Training team, several ELIXIR Nodes (Belgium, Italy and UK) and CyVerse in United States.

##

**Key Points**

- EMBL-ABR was launched on February 2016 and includes 10 nodes distributed across Australia.
- EMBL-ABR has a direct connection with the user communities by including biological domain experts across its structure, including its International Advisory group.
- EMBL-ABR activities and remit are spread across six key areas: Data, Tools, Platforms, Standards, Compute and Training.
- EMBL-ABR has collected during 2016 information about bioinformatics training events and materials produced in Australia and Tools created in Australia, by federation of existing solutions (Biotools.org and STM) rather than recreating such efforts from scratch.
- EMBL-ABR is fostering and promoting awareness of Australian bioinformatics and its bioscience overall across a variety of international institutions and collaborations.
